# Trust Transitivity in Social Networks

**DOI:** 10.1371/journal.pone.0018384

**Published:** 2011-04-05

**Authors:** Oliver Richters, Tiago P. Peixoto

**Affiliations:** Institut für Festkörperphysik, Technische Universität Darmstadt, Darmstadt, Germany; University of Maribor, Slovenia

## Abstract

Non-centralized recommendation-based decision making is a central feature of several social and technological processes, such as market dynamics, peer-to-peer file-sharing and the web of trust of digital certification. We investigate the properties of trust propagation on networks, based on a simple metric of trust transitivity. We investigate analytically the percolation properties of trust transitivity in random networks with arbitrary in/out-degree distributions, and compare with numerical realizations. We find that the existence of a non-zero fraction of *absolute trust* (i.e. entirely confident trust) is a requirement for the viability of global trust propagation in large systems: The average pair-wise trust is marked by a discontinuous transition at a specific fraction of absolute trust, below which it vanishes. Furthermore, we perform an extensive analysis of the Pretty Good Privacy (PGP) web of trust, in view of the concepts introduced. We compare different scenarios of trust distribution: community- and authority-centered. We find that these scenarios lead to sharply different patterns of trust propagation, due to the segregation of authority hubs and densely-connected communities. While the authority-centered scenario is more efficient, and leads to higher average trust values, it favours weakly-connected “fringe” nodes, which are directly trusted by authorities. The community-centered scheme, on the other hand, favours nodes with intermediate in/out-degrees, in detriment of the authorities and its “fringe” peers.

## Introduction

Several social and technological systems rely on the notion of trust, or recommendation, where agents must make their decision based on the trustworthiness of other agents, with which they interact. One example are buyers in markets [1], who may share among themselves their experiences with different sellers, or lenders which may share a belief that a given borrower will not be able to pay back [2]. Another example are peer-to-peer file-sharing programs [3], which often must know, without relying on a central authority, which other programs act in a fair manner, and which act selfishly. In the same line, an even more direct example is the web of trust of digital certification, such as the Pretty Good Privacy (PGP) system [4], [5], where regular individuals must certify the authenticity of other individuals with digital signatures. In all these systems, the agents lack global information, and must infer the reliability of other agents, based solely on the opinion of trusted peers, thus forming a network of trust. In this paper, we present an analysis of trust propagation based on the notion of *transitivity*: If agent 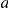 trusts agent 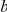, and agent 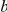 trusts agent 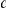, then, to some extent, agent 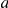 will also trust agent 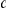. Based on this simple concept, we define a trust metric with which the reliability of any reachable agent may be inferred. Instead of concentrating on the minutiae of trust propagation semantics, we focus on the topological aspect of trust networks, using concepts from network theory [6]. Using random networks as a simple model, we investigate the necessary conditions for trust to “percolate” through an entire system. We then apply the concepts introduced to investigate in detail the PGP web of trust, possibly the best “real” example of a trust propagation system, which is completely accessible for investigation. We focus on the role of the strongly connected nodes in the network — the so called *trust authorities* — which represent a different paradigm of trust delegation, in comparison to the decentralized community-based approach, which is also heavily present in the network.

This paper is divided as follows. In section 1 we define the trust metric used; in section 2 we consider the problem of trust percolation in random networks with different trust weight distributions. In in section 3 we turn to the analysis of the PGP network, and provide an extensive analysis of its topology, and of trust propagation according to different trust distribution scenarios. Finally, we provide some final remarks and a conclusion.

## Analysis

### 1 Trust metric

Trust is the measure of belief that a given entity will act as one expects. It is often associated with positive, desirable attributes, but it may not always be the case (e.g. one may have trust that someone will act undesirably). Humans use trust to make decisions when more direct information is unavailable. In general, humans will decide their level of trust based on arbitrary, heuristic rules, since there is no formal consensus on how to evaluate trust. We will deliberately avoid the detailed formalization of these rules, and instead rely on two simplifications: 1. We will treat trust simply as a probability that a given assessment about an agent is true or false (e.g. fair/reliable or not); 2. We further assume that this belief is *transitive*, i.e. if agent 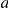 trust agent 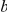, which in turn trusts agent 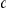, then 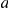 will also trust 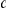, to some extent. This makes trust propagation easier to analyse, while retaining its most intuitive properties.

**Figure 1:**
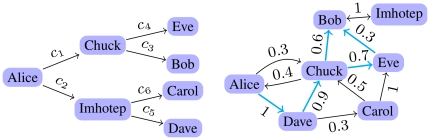
Examples of trust networks. **Left:** A directed tree. **Right:** A more realistic example. The edges in blue are the ones which contribute to the value of trust from Bob to Alice, according to Eq. 7.

**Figure 2:**
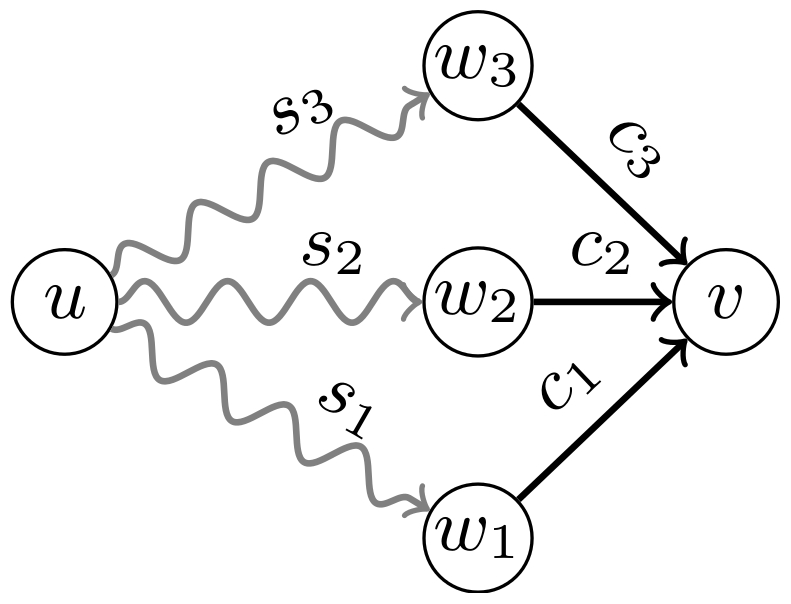
Illustration of the paths used to calculate 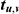 according to Eq. 7. The vertices 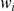 are the in-neighbours of 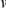, and the values 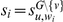 are the values of best trust (Eq. 1) from 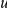 to 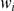, with vertex 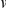 removed from the graph.

We will consider a system of 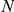 agents which form a directed trust network: Each agent 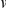 (represented by a vertex, or node) has a number of interactions (represented by directed edges, or links) with other agents 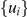 for which a value 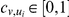 of *direct trust* is defined *a priori*, and which can be interpreted as a probability. This value represents a direct experience agent 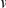 had with 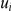, which is not inferred from any other agent. We note that this value fully reflects the directed nature of the network, so that if there is also an edge 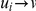, the value of 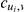 is in independent of 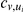 — in other words, direct trust does not need to be reciprocal. Additionally, we do not assume that there is an inherent self-loop from each vertex to itself. If a self-loop 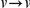 exists, we do not ascribe any special meaning to the diagonal element 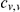, which can be arbitrarily chosen just as any other direct trust value. We then define the *inferred trust* 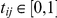 from agent 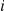 to any agent 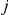, which is somehow based on the values of 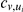, which is somehow based on the values of 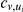. In a simple situation where there is only one possible path between any two given nodes (e.g. the network is a directed tree, as the example on the left in Fig. 1), one could simply multiply the values of 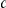 along the single path to obtain 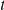, e.g. 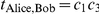, in the example of Fig. 1 (throughout this work, a path is always considered to be *self-avoiding*, i.e. no edge or vertex is visited twice). In general, however, the situation may be more complicated, as in the example on the right of Fig. 1, where there is a variety of possible (often “contradictory”) transitive paths between most pairs of nodes. Perhaps the simplest way of defining a trust metric would be to consider only the *best* transitivity path between two nodes, i.e., the one where the trust transitivity is maximum, 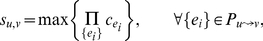
(1)


where 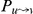 is the set of all paths from 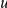 to 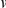, 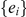 is the set of edges in a given path, and 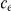 is the direct trust associated with a given edge (if there is no path from 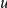 to 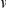, we consider the value of 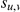 to be zero. Additionally, we consider the diagonal values of best trust to be equal to one, i.e. 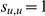). This definition is an attractive one, since it corresponds directly to the concept of minimum distance on weighted graphs, which is defined as the sum of weights along the path with the smallest sum. This is easily seen by noticing that 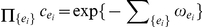, with 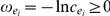 being the edge weights (with the special value of 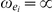 if 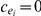). However, it is clear that this approach leads to an optimistic bias, since the best path obviously favors large values of trust, and uses only a small portion of the information available in the network. As an illustration consider the network on the right of Fig. 1, where the value of 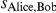 is 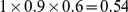, via Dave and Chuck. However, if Chuck is directly consulted, the transitivity drops to 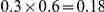. In principle, there is no reason to prefer any of the two assessments over the other. One may attempt to rectify this by considering instead *all* possible paths between two nodes,

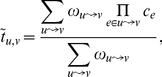
(2)


where 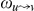 is a weight associated with a given path 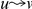. It should be chosen to minimize the effect of a very large number of paths with very low values of trust, without introducing an optimistic bias on the final trust value. One apparently good choice is to consider the transitivity value of the path itself, but not including the last edge, 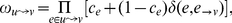
(3)


where 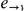 is the last edge in the path, and 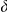 is the Kronecker delta. The usage of Eq. 3 is apparently appropriate since it not only avoids a bias in the final value of 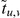, but also 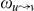 has a simple interpretation as being the value of trust on the *final* recommendation, which is completed by the last edge. While this may seem reasonable, and uses all available information in the network, it has two major drawbacks: 1. It is very computationally costly to consider all possible paths between two nodes, even in moderately sized networks. It would represent an unreasonable effort on part of the agents to use all this information. 2. Computed as in Eq. 2, the value of 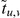 has the unsettling behaviour of tending to zero, whenever the number of paths become large (as they often are), even when paths are differently weighted. Consider a simple scenario where the network is a complete graph, i.e. all possible edges in the network exist, and all of them have the same direct trust value 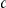. Since there are 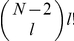 paths of length 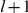 between any two vertices, the value of inferred trust between any two nodes can be calculated as 
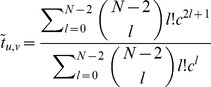
(4)

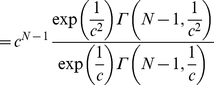
(5)

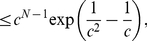
(6)


where 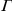 is the upper incomplete gamma function, from which it is easy to see that 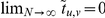 for 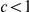. This is an undesired behavior, since one would wish that such highly connected topologies (which often occur as subgraphs of social networks, known as *cliques*) would result in *higher* values of trust. In order to compensate for this one would have to use a more aggressive weighting of the possible paths. We propose the following modification, which combines some features of both previous approaches: Instead of considering all possible paths, we consider only those with the largest weights to all the in-neighbours of the target vertex, as shown in Fig. 2. This leads to a trust metric defined as 
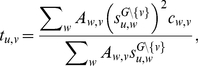
(7)


where the path weights are the best trust transitivity to the in-neighbours, 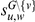, which are calculated after removing the target vertex from the graph (so that it cannot influence its own trust), and 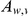 is the adjacency matrix, defined as

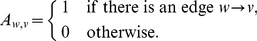
(8)


Like for 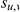, we assume that 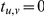 if there is no path from 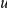 to 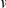, and 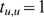, for any 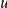. We note that the term 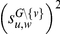 comes from the multiplication of the trust being averaged, 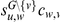, and its corresponding weight 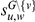. We call this trust metric *pervasive trust*, and it corresponds to the intuitive strategy of searching for the nodes with a direct interaction with the target node (the final arbitrators), and weighting their opinions according to the best possible trust transitivity leading to them. It can be seen that this definition does not suffer from the same problems of Eq. 2, again by considering the same complete graph example, with uniform direct trust 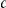. Since in this situation every target vertex has 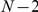 in-neighbours different from the source, and the shortest path to each of these in-neighbours is of length one, the value of pervasive trust can be easily calculated as

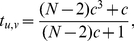
(9)


for 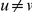, which converges to 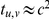 for 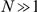. Thus the indirect opinions with value 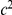 dominate the direct trust value 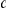, but the inferred value does not vanish, as with the definition of Eqs. 2 and 3. Considering again the example on the right of Fig. 1, we obtain the value 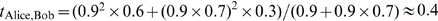, from the edges outlined in blue in the figure. Additionally, the definition of pervasive trust works as one would expect in the trivial example on the left of Fig. 1, where 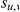 and 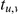 have the same values.

We note that the numerical computation of 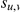 can be done by using Dijkstra's shortest path algorithm [7], [8], which has a complexity of 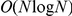. Thus the entire matrix 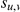 can be calculated in 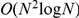 time. The same algorithm can be used to calculate 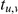, but since each target vertex needs to be removed from the graph, and thus a new search needs to be made for each different target, this results in 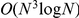 time. It is possible to improve this by performing searches in the *reversed* graph, i.e., for each target vertex 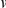, the contribution to 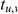 from all sources 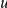 can be calculated simultaneously, after 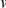 is removed, by performing a single reversed search from each of the in-neighbours of 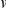 to each source 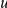. This way, the entire 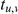 matrix can be computed in 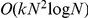 time (where 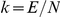 is the average in/out-degree of the network), which is comparable to the computation time of 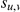 for sparse graphs.

#### 1.1 Comparison with other trust metrics

Other trust metrics have been proposed in the literature, mainly by computer scientists, seeking to formalize the notion of trust in peer-to-peer computer systems. Some are quite detailed, like the usage of subjective logic by Jøsang et al [9], and others are comparable with the simplistic approach taken in this work, such as Eigentrust [3] and more recently TrustWebRank [10]. These last metrics are based on the notion of *feedback centrality* [8], which is usually defined as some linear system involving the adjacency matrix. The Eigentrust metric requires the trust network to be a stochastic matrix (i.e. the sum of the trust values of the out-edges of all vertices must sum to unity) and the inferred trust values are given by the steady state distribution of the corresponding Markov chain (i.e. the left eigenvector of the stochastic matrix with unity eigenvalue, hence the name of the metric). Thus the inferred trust values are *global* properties, independent of any source vertex (i.e. non-personalized), which is non-intuitive. Additionally, the requirement that the trust network is stochastic means that only *relative* values of trust are measured, and the absolute information is lost. Furthermore, such an approach is strongly affected by the presence of loops in the network, which get counted multiple times, which is also non-intuitive as far as trust transitivity is concerned. The metric TrustWebRank [10] tries to fix some of these problems by borrowing ideas from the PageRank [11] algorithm, resulting in a metric which also requires a stochastic matrix, but is personalised. However, in order for the algorithm to converge, it depends on the introduction of an *damping factor* which eliminates the contribution of longer paths in the network, independently of its trust value. This is an *a priori* assumption that these paths are not relevant, and may not correspond to reality. Additionally, the strange role of loops in the network is the same as in the Eigentrust metric. However, since there is no consensus on how a trust propagates, and the notion of trust lacks a formal, universally accepted definition, in the end there is no “correct” or “wrong” metric. We only emphasize that our approach is derived directly from the simple notion of trust transitivity, is easy to interpret, propagates *absolute* values of trust, and makes no assumption whatsoever about the network topology, and direct trust distribution.

## Results

### 1 Trust percolation

Trust transitivity is based on the multiplication of direct trust values, which may tend to be low if the paths become long. Therefore, it is a central problem to determine if the trust transitivity between two randomly chosen vertices of a large network vanishes if the system becomes very large. This provides important information about the viability of trust transitivity on large systems. As a simple network model, we will consider random directed networks with arbitrary in/out-degree distributions [12]. We will also suppose that the direct trust values in the range between 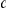 and 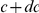 will be independently distributed with probability 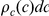, where 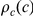 is an arbitrary probability density function (PDF). The objective of this section is to calculate the average best trust transitivity 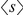, given by Eq. 1, and the average pervasive trust 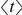, Eq. 7, between randomly chosen pairs of source and target vertices. In random networks, the value of average pervasive trust will be given simply as 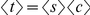, since the best paths to the in-neighbours of a given vertex are uncorrelated, and the probability that they pass through the node itself tend to zero, in the limit of large network size. Therefore we need only to concern ourselves with the average best trust transitivity 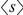.

Directed networks are composed of components of different types and sizes: For each vertex there will be an *out-component*, which is the set of vertices reachable from it, and an *in-component*, which is the set of vertices for which it is reachable. A maximal set of vertices which are mutually reachable is called a *strongly connected component*. Random graphs often display a phase transition in the size and number of these components: If the number of edges is large enough, there will be the sudden formation of a giant (in-, out-, strongly connected) component, which spans a non-vanishing fraction of the network [6], [12]. The existence of these giant components is obviously necessary for a non-vanishing value of trust to exist between most vertices, but it is not sufficient, since it is still necessary that the multiplication of direct trust values along most shortest paths do not become vanishingly small. As an illustration, consider a sparse graph (i.e. with finite average in/out-degree), with a arbitrary in/out-degree distributions. In the situation where there is a sufficiently large giant out-component in the graph, the average shortest path from a randomly chosen root vertex to the rest of the network is given approximately [12] by
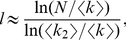
(10)


independently of the out-degree distribution (as long as 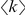 and 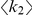 are finite positive), where 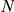 is the number of vertices, 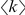 is the average out-degree and 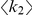 is the average number of second out-neighbours, and it is assumed that 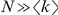 and 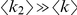 (an analogous expression for the distance from the entire network to a randomly chosen *target* can be obtained by replacing 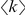 and 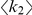 with the average in-degree and second in-neighbours, 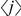 and 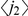 respectively). Since the edges are weighted, the average length of the best paths can differ from 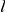, but can never be smaller. Thus, an upper bound on the average best trust is given by 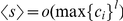, where 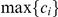 is the maximum value of direct trust in the network. In the situation where 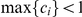, we have that 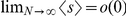, since 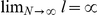. Therefore, if there are no values of 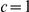 in the network, the average trust will always be zero in sparse networks. The only possible strategies for non-vanishing values of average trust is either to have a non-zero fraction of 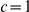 (which we will call *absolute trust*), or for the network to be dense, such that 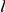 remains finite for 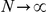.

**Figure 3:**
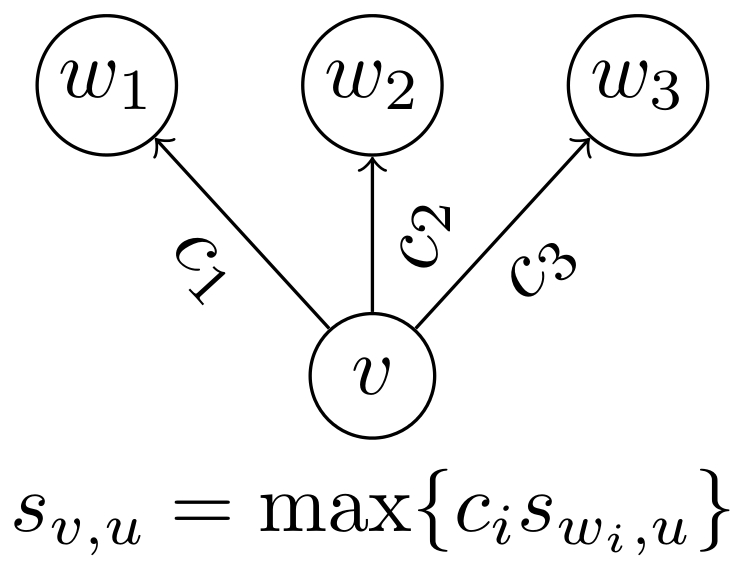
Neighbourhood of vertex 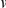 with out-neighbours 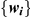 with direct trust 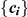. The best trust from 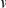 to an arbitrary vertex 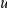, 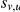, is given as a function of 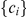 and 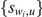, according to Eq. 11.

With the above consideration in mind, we now move to calculate the average trust transitivity values. We will obtain a self-consistency condition for the distribution of best trust transitivity values, by describing the direct neighbourhood of a single vertex, similarly to what was done in [12] to obtain the distribution of component sizes. For simplicity, we will consider only the situation where the in- and out-degrees of the vertices are uncorrelated. The approach is based on the following observation. Consider two randomly selected vertices, 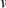 and 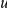, and the best trust from 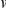 to 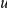, 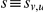, which is distributed according to a PDF 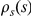. Let 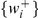 be the set of out-neighbours of 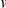 (we assume that the probability of 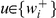 vanishes for 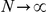), with direct trust values 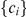, as illustrated in Fig. 3. It is clear that the value of 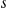 can be written as a function of the best trust from each out-neighbour 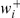 to 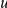, 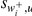, as
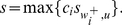
(11)


We note that an analogous equation can be obtained in the opposite direction, by considering the in-neighbours 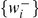 of 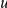, with direct trust values 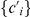, and their best trust values 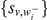, 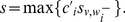
(12)


**Figure 4:**
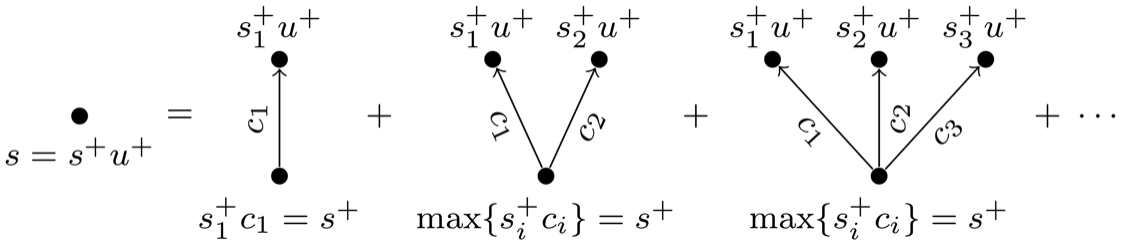
Schematic representation of the self-consistency for 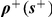 in Eq. 14. Each term corresponds to the probability of the vertex having a given number of out-neighbours, and the maximum best trust transitivity being equal the desired value.

Each equation above can be used to establish a self-consistency equation for appropriately defined auxiliary distributions, which can be combined to obtain 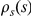, as will be explained below. The main intuitive notion which will be explored is that on uncorrelated random graphs, the properties of a given vertex and its out/in-neighbours should be the same on average. Therefore, certain distributions associated with variables on the left hand side of Eqs. 11 and 12, are also associated with variables which appear on the right hand side. In order to express the self-consistencies in detail, we need to introduce two auxiliary variables 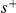 and 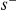 and their PDFs 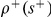 and 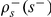. The PDF 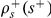 will be associated with Eq. 11 and the out-degree distribution, and 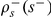 with Eq. 12 and the in-degree distribution. Without loss of generality, we describe only the self-consistency for 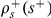 in detail, since the development for 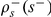 can be obtained in an entirely analogous fashion, by replacing the out-degree with the in-degree. In order to transform Eq. 11 into a self-consistency equation, we need to define yet another auxiliary distribution, 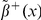, which is the cumulative probability that 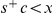, with 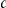 being the direct trust, distributed according to 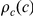, given by 
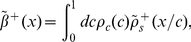
(13)


where 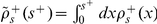 is the cumulative distribution of 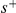. Now, if we suppose that the best trust values 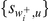 from the out-neighbours 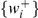 of 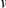 are distributed according to 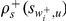, we obtain that the cumulative probability that the right hand side of Eq. 11 is less than 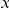 is given by 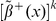, where 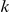 is the out-degree of vertex 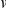. A full self-consistency equation for 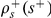 can be obtained by supposing that the value of 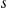 is distributed according to the same distribution as 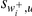, and considering all the possible out-degrees and their respective probabilities, as follows (see Fig. 4): The cumulative probability that 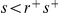, where 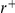 is an arbitrary value which will not influence the self-consistency, will be given by the sum of the probabilities that vertex 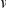 has out-degree 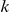 multiplied by the cumulative probability that 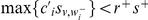 for all 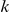 out-neighbours. Concisely, this can be expressed as 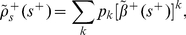
(14)


where 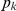 is the out-degree distribution. Note that while Eq. 14 is a self-consistency condition from which 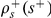 can be obtained (given 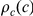 and 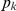), it cannot be used to obtain 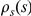 directly, because of the arbitrary value 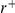 which does not influence Eq. 14. We note however that, as mentioned previously, Eq. 12 can be used to obtain an equation for 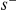 and 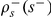 which is entirely analogous to Eq. 14, with 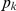 replaced by the in-degree distribution 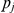. This equation is also not affected by an analogous arbitrary value 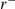. Since we have two self-consistency relationships which are defined up to two arbitrary values, they can be used to complement each other by formulating the ansatz that 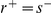 and 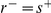, which leads to
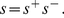
(15)


With this connection it is possible to obtain 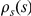 from 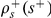 and 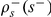 simply as

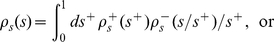
(16)

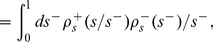
(17)


and the average 
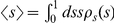
 more directly as

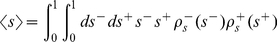
(18)

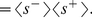
(19)


By rewriting Eq. 14 in terms of the generating functions of the in- and out-degree distributions,
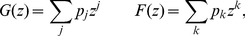
(20)


one obtains the self-consistency equations in a more compact form,

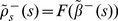
(21)

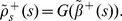
(22)


These are integral equations, for which there are probably no general closed form solutions. However, it is possible to solve them numerically by successive iterations from an initial distribution, which we chose as 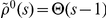, where 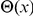 is the Heaviside step function. From the numerical solutions the average values can be obtained as 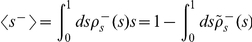 (where the last expression is obtained by integration by parts), and in analogous fashion for 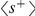. The average value of best trust transitivity 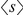 is then given by Eq. 19.

We turn now to the conditions necessary for non-vanishing average trust transitivity. Both Eqs. 21 and 22 accept the trivial solution 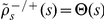, which corresponds to 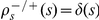, i.e. the average best trust is zero. As discussed previously, for other solutions to be possible, we need to consider a non-vanishing fraction of edges with absolute trust 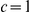 in the network. Here we will consider direct trust distributions of the form, 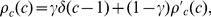
(23)


which correspond to a fraction 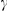 of edges with 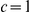, and a complementary fraction 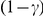 with 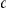 given with probability density 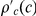. We will consider two different versions of 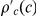: A uniform distribution 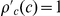, and a single-valued distribution 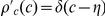, with 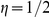. We will use two different in/out-degree distributions, the Poisson and Zipf, and their respective generating functions, 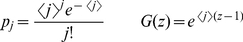
(24)

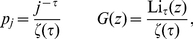
(25)


where 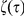 is the Riemann 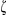 function, and 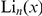 is the 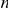
th polylogarithm of 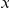. For simplicity, we will consider only the situation where 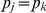, and both the in-degree 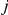 and the out-degree 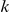 are independently distributed.

**Figure 5:**
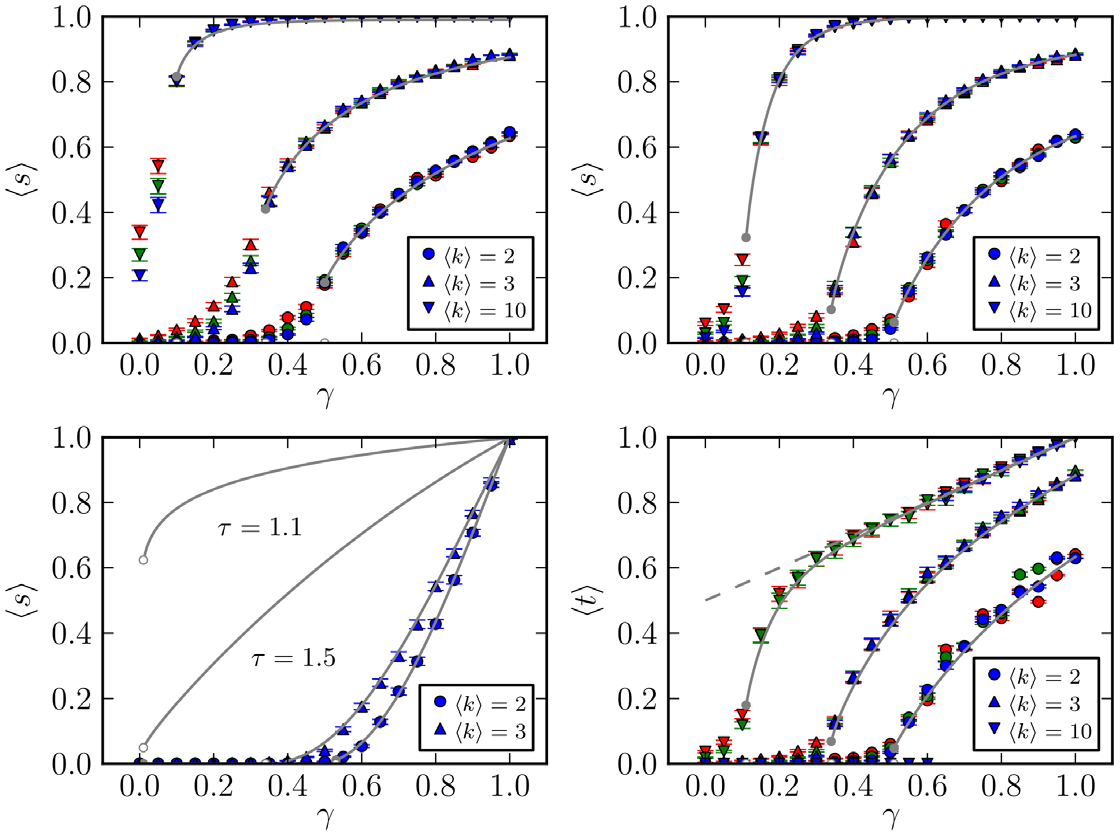
Average values of best trust 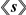 and pervasive trust 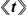 as a function of the fraction of edges with absolute trust 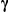. **Top left:** Networks with Poisson in- and out-degree distributions, and uniform trust distribution. **Top right and bottom right:** Poisson distribution, and single-valued trust distribution. **Bottom left:** Zipf distribution, and single-valued trust distribution. Solid lines correspond to analytical solutions, and symbols to numerical realizations of several networks of different sizes: 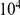 (red), 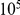 (green) and 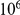 (blue) nodes. The dashed line shows the average direct trust 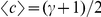.

In Fig. 5 are plotted the values of 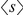 and 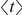, as a function of 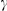, for the different distributions. It is also compared with numerical computations on actual network realizations of different sizes. The main feature observed is a first-order transition from vanishing trust to positive trust, at specific values of 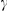. This is an interesting feature, since it seems at first to be at odds with traditional percolation theory, which predicts a second-order transition. However, we point out that the order parameter 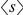 is very different from what usually characterises a percolation transition, namely the relative size of the largest connected component. Although we used a similar technique to obtain 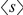, there is no *a priori* reason to expect its transition to be continuous, and indeed it seems not to be the case. It is possible, however, to identify a very direct connection to the conventional percolation transition, given by the values 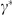 where the transition for 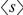 occurs: If one considers the subgraph composed of all the vertices and only the edges with 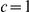, it can be easily concluded that this subgraph is a random graph on its own, since the values of 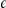 are randomly distributed on the edges. Its in/out-degree distributions will in general be different than for the complete graph, with an average given by 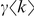. For a Poisson distribution, the usual percolation transition occurs when the average in/out-degree is one [12], which, for the 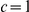 subgraph, corresponds to 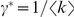. These are indeed the transition points observed for 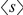, when in/out-degree distributions are Poisson. Therefore, the transition values 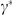 correspond exactly to the critical values of the formation of a giant component of the subgraph composed only of edges with 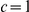. It is worth observing that on finite graphs, the average trust does not vanish very rapidly, and is still non-zero for relatively large networks with 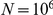 vertices, even when 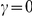. This seems to be simply a finite size effect, intensified by the the so-called small-world property, where the average shortest path scales slowly as 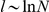, as in Eq. 10. As can be seen in in Fig. 5, for some of the networks of size up to 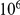 vertices, the values of 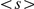 below the transition have not yet converged to a value which no longer depends on N, which clearly indicates a finite size effect. This is further corroborated by the values of 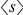 for 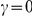, which are sometimes above zero, even though in this situation they *must* be equal to zero in the limit 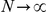, as explained in detail previously. This very strong finite size effect means also that in practical situations where networks are large but finite, 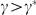 it is not a strictly necessary condition for system-wide trust propagation.

Another interesting feature is the behaviour of the average trust in graphs with Zipf in/out-degree distribution. There, the transition to positive trust is of second order, and the critical points are also 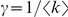. Additionally, the values of average trust are smaller than in networks with Poisson in/out-degree distribution and the same average in/out-degree, for intermediary values of 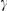 after the transitions. This is due to the smaller path multiplicity of graphs with scale-free distribution: Even though the average shortest path length is smaller in such graphs, the number of alternative paths is also smaller, due to the dominance of vertices with smaller in/out-degree. Thus, if the shortest path happens to have a small trust value, there will be a higher probability there will not be an alternative path. In Fig. 5 it is shown also the average best trust for 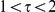, for which the average in/out-degree diverges. For such dense networks, the values of 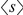 are above zero for all values of 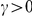, which means that any small (but existing) fraction of edges with 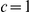 can be used by most shortest paths in this case.

### 2 The Pretty Good Privacy (PGP) Network

In this section we investigate trust propagation on the Pretty Good Privacy (PGP) network. In a broad manner PGP (or more precisely the OpenPGP standard [13]) refers to a family of computer programs for encryption and decryption of files, as well as data authentication, i.e. generation and verification of digital signatures. It is often used to sign, encrypt and decrypt email. It implements a scheme of public-key cryptography [14], where the keys used for encryption/decryption are split in two parts, one private and one public. Both parts are related in way, such that the private key is used exclusively for decryption and creation of signatures, and the public key only for encryption and signature verification. Thus any user is capable of sending encrypted messages and verifying the signature of a specific user with her public key, but only this user can decrypt these messages and generate signatures, using her private key, which she should never disclose. The public keys are usually published in so-called key servers, which mutually synchronize their databases, and thus become global non-centralized repositories of public keys. However, the mere existence of public key in a key server, associated with a given identity (usually a name and an email address) is no guarantee that this key really belongs to the respective person, since there is no inherent verification in the submission process. This problem is solved by the implementation of the so-called *web of trust* of PGP keys, whereby a user can attach a signature to the public key of another user, indicating she trusts that this key belongs to its alleged owner. The validity of a given key can then be inferred by transitivity, in a self-organized manner, without the required presence of a central trust authority. As such, this system represents an almost perfect example of a trust propagation through transitivity.

As a rule, key signatures should only be made after careful verification, which usually requires the two parties to physically meet. Such a requirement transforms the web of trust into a snapshot of a global social network of acquaintances, since the vast majority of keys correspond to human users, which tend to sign keys of people with which they normally interact. There is also a tendency to sign keys (upon verification) from people which do not belong to a close circle of acquaintances, with the sole purpose of strengthening the web of trust with more connections. This tendency is well reflected by the so-called “key signing parties”, where participants meet (usually after a large technological conference) to massively sign each other's keys [15]. Thus the structure of the PGP network reflects the global dynamics of self-organization of human peers in a social context.

This section is divided in two parts. In the first part we present some aspects of the topology and temporal organization of the network. In the second part we analyze the trust transitivity in the network, in view of the trust metric we discussed previously.

#### 2.1 Network topology

The PGP network used in this work was obtained from a snapshot of the globally synchronized SKS key servers (available at http://key-server.de/dump/) in November 2009. It is composed of 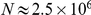 keys and 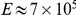 signatures with a very low average in-degree of 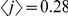. This means that many keys are isolated and contain no signatures. Therefore we will concentrate on the largest *strongly connected component*, i.e. a maximal set of vertices for which there is a path between any pair of vertices in the set. The number of vertices 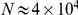 in this component is much smaller, but the network is much denser, with on average 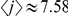 signatures per key (see summarized data in table 1). It represents the *de facto* web of trust, since the rest of the network is so sparsely connected that no trust transitivity can be inferred from it. We note that keys may have multiple “subkeys” which correspond to different identities (usually different email addresses from the same person) and which can individually sign other subkeys. For simplicity, in this work we have collapsed subkeys into single keys, and possible multiple signatures into a single signature. We have also discarded invalid, and revoked keys and signatures.

**Table 1:** Summary of statistics for the whole PGP network (above) and the largest strongly connected component (below).

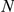	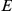	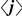	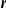	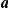	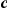
2513677	703142	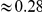	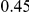	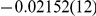	0.02321(9)
39796	301498	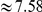	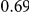	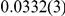	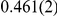

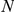
 is the number of vertices (keys), and 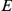 is the number of edges (signatures), 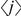 is the average in-degree, 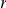 is the average reciprocity, 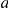 is the assortativity coefficient and 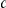 is the average clustering coefficient.

**Figure 6:**
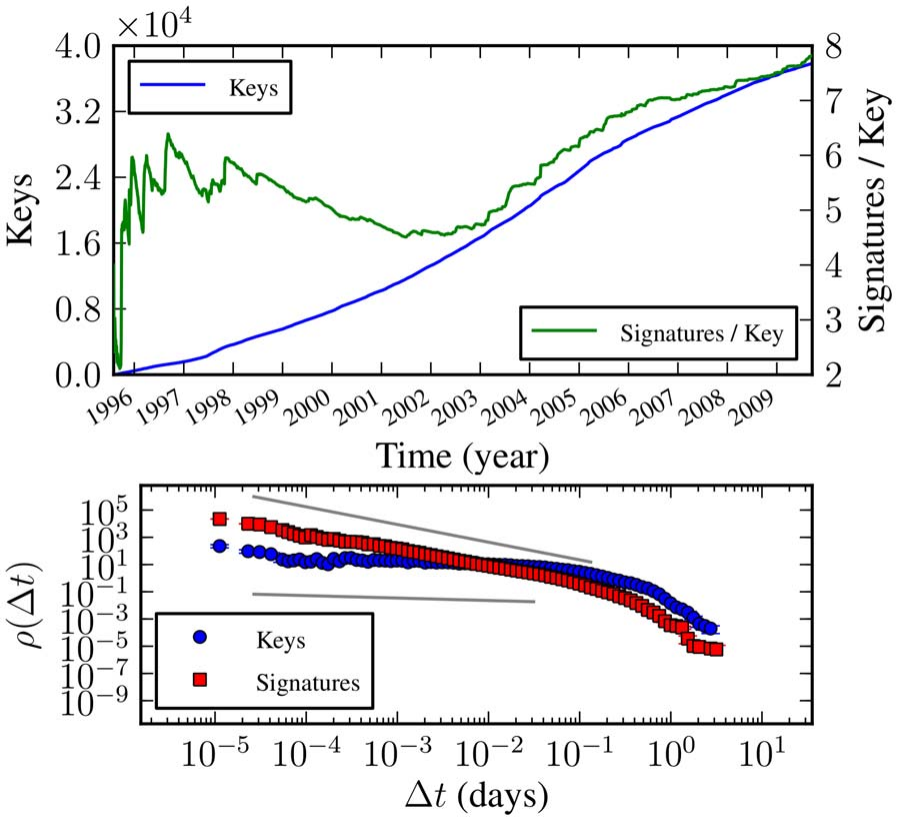
Number of keys and signatures as a function of time for the strongly connected component of the PGP network, and waiting time distribution between new keys and signatures. The straight lines are power-laws 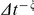, with 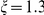 (top) and 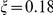 (bottom).

The number of keys and signatures in the strongly connected component has been increasing over time, as shown in Fig. 6. The number of keys (which are now valid) was approximately the same for some time and then slightly decreased for a period up to around 2002, and has been increasing with an approximately constant rate since then. We note that the number of keys may decrease, since keys can expire or be revoked. The number of signatures, on the other hand, seems to be increasing with an accelerated rate, with an approximately constant acceleration, which is similar to the rate of growth of the number of keys. This means that the average in/out-degree of the network is increasing with time, as can be seen in Fig. 6. Keys and signatures grow in an organized manner, as shown by the waiting time distribution between the creation of two subsequent keys or signatures, as shown in Fig. 6. These distributions are broad for several orders of magnitude, from the order of seconds to days, approximately following a power-law in this region. The fact that keys and signatures are often created only seconds apart, and the waiting time distribution lacks any discernible characteristic scale, except for a cut-off at large times (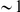 day), shows that the network does not grow in a purely random fashion (which would generate exponentially-distributed waiting times, as in an homogeneous Poisson process. If the Poisson process is non-homogeneous, with a constantly accelerating rate, the waiting times would follow instead a Weibull distribution, which also has an exponential tail), and serves as a signature of an underlying organized growth process.

We will characterize the topology of the network by its in/out-degree distribution and nearest-neighbours in/out-degree correlations, as well as other standard network measures such as clustering [16], reciprocity [17] and community structure [18]. We will pay special attention to the most highly connected vertices, some of which correspond to so-called *certificate authorities* and display a distinct connectivity pattern, which has a special meaning for trust propagation.

**Figure 7:**
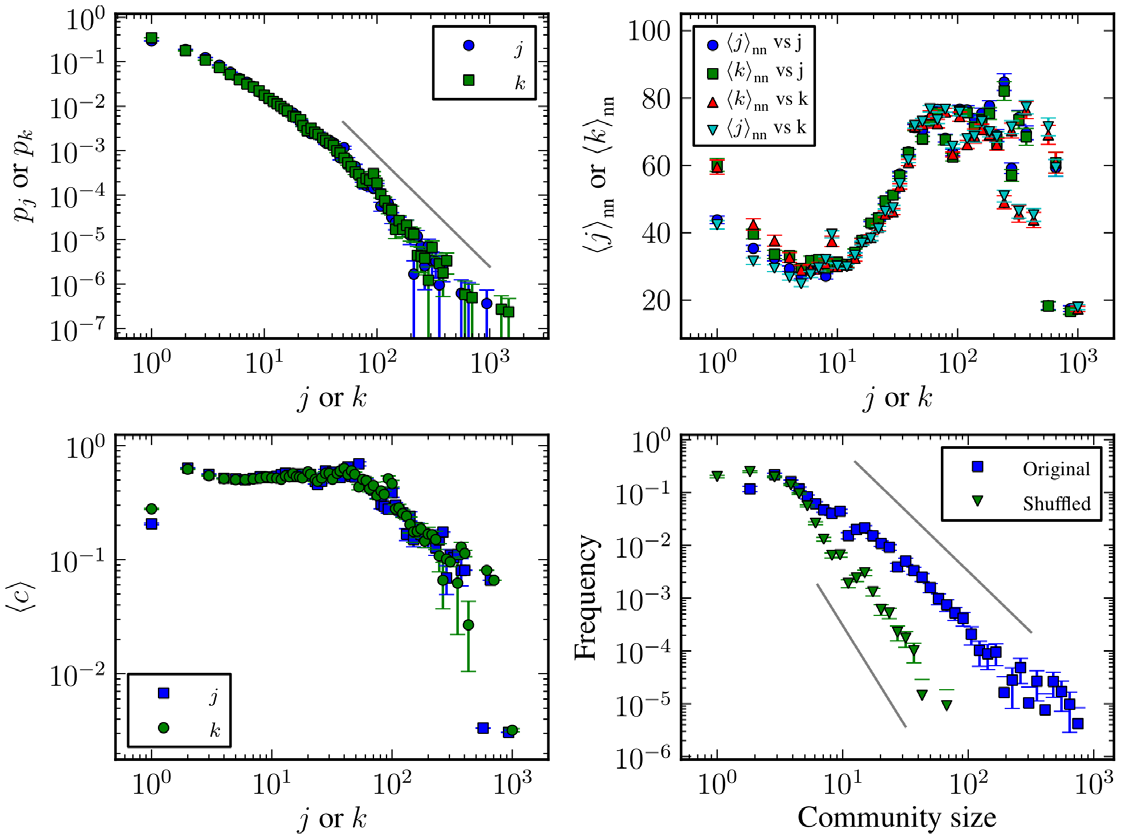
Several statistical properties of the PGP Network. **Top left:** In- and out-degree distributions, 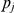 and 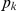 respectively. The solid line corresponds to a power-law with exponent 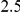. **Top right:** Average in- and out-degree of the nearest out-neighbours, as a function of the in- and out-degree. **Bottom left:** Average lustering coefficient as a function of in- and out-degree. **Bottom right:** Distribution of community sizes, for the unmodified and shuffled versions of the network. The solid lines correspond to power-laws with exponent 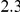 (top) and 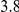 (bottom).

**Figure 8:**
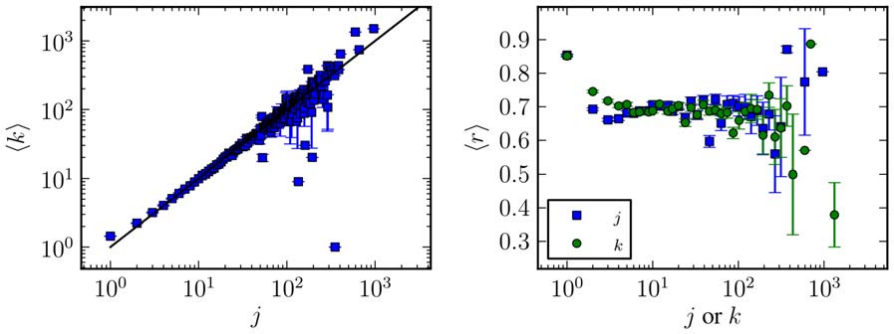
Reciprocity statistics of the PGP network. **Left:** Average out-degree as a function of the in-degree of the same vertex. **Right:** Average edge reciprocity, as a function of the in or out-degree of the source vertex.

The network has very heterogeneous in/out-degree distributions, as can be seen in Fig. 7, with some keys having on the order of 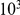 signatures. They are possibly compatible with a power-law with exponent 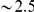 for large in/out-degrees, but the distributions are not broad enough for a precise identification. The number of signatures on a given key (the in-degree) and the number of signatures made by a the same key (the out-degree) are strongly correlated, as can be seen in Fig. 8, which shows the average out-degree 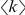 as a function of the in-degree 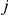. This is explained by the high reciprocity of the edges in the network, i.e. if a key 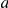 signs a key 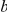, there is a very high probability that key 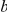 signs key 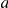 as well. This is easy to understand, since key verification usually requires physical presence, and both parties take the opportunity to mutually verify each other keys in the same encounter. The edge reciprocity [17] is quantified as the fraction 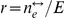, where 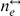 is the number of reciprocal edges and 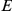 is the total number of edges in the network. The PGP network has a high value of 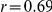. The reciprocity is distributed in a slightly heterogeneous fashion across the network, as is shown in Fig. 8, where is plotted the average reciprocity of the edges as a function of the in- and out-degrees of the source vertex. It can be seen that the keys with very few signatures tend to act in a very reciprocal manner, whereas the more prolific signers receive less signatures back. This heterogeneity is further amplified when one considers the in/out-degree correlation between nearest-neighbours, as shown in Fig. 7, where it is plotted the average in- and out-degree, 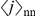 and 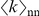, of the nearest out-neighbours of the vertices in the network, as a function of the in- and out-degree of the source vertex, 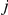 and 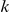. The in/out-degree correlation shows an *assortative* regime for intermediary in/out-degree values (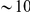 – 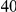), meaning that vertices with higher in/out-degrees are connected preferentially with other vertices with high degree, but also some *dissortative* features for vertices with very high and very low in/out-degrees, where vertices with low in/out-degree are connected preferentially with vertices with high in/out-degree, and *vice versa*. This mixed connectivity pattern leads to a very low scalar assortativity coefficient [19] of 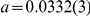, which is an unusually small value for social networks [20] (the scalar assortativity coefficient is defined for an undirected graph as 
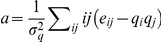
 where 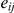 is the fraction of edges that connect vertices of degrees 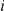 and 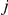, 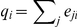 and 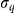 is the standard deviation of the distribution 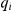. This definition yields values in the range 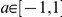, with 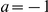 for networks which are maximally dissortative, and 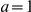 for maximally assortative. For the PGP network, the direction of the edges was ignored in the calculation of 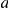). These differences become more clear when one investigates more closely the keys with the largest in-degree in the network, as it is shown in table 2. As with the rest of the network, most of the largest keys belong to individuals, with the exception of the first and third keys with the most signatures, which belong to entities. These entities are known as *certificate authorities* and are created by organizations with the intent of centralizing certification. The largest authority is the community-driven CAcert.org which issues digital certificates of various kinds to the public, free of charge (See the CAcert.org website: http://cacert.org). The second largest authority is the German magazine c't, which initiated a PGP certification campaign in 1997 (A second, older c't key is also still among the largest hubs, with 289 signatures. See http://www.heise.de/security/dienste/Krypto-Kampagne-2111.html for more details). These authorities interact with individuals in a different manner, acting as a central mediator between loosely connected peers. This is evident by the low clustering coefficient (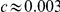), which is one order of magnitude lower than the other (human) hubs (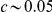 – 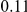), and the average in-degree of their out-neighbours, which is also significantly smaller than their human counterparts (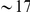 vs. 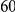 – 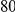, respectively). These different patterns represent distinct paradigms of trust organization: Authority vs. Community-based; each with its set of advantages and disadvantages. An authority-based scenario relies on few universally trusted vertices which mediate all trust propagation. In this way, the responsibility of key verification is concentrated heavily on these vertices, which reduces the total amount of verification necessary, and is thus more efficient. The most obvious disadvantage is that the authorities represent central points of failure: if an authority itself is not trusted, neither will be the keys it certifies. Additionally, this approach may increase the probability of forgery, since only one party needs to be deceived in order for global trust to be achieved. The complementary scenario is the community-based approach, where densely-connected clusters of vertices provide certification for each other. This obviously requires more diligence from the participants, but has the advantage of larger resilience against errors, since the multiplicity of different paths between vertices is much larger. In the PGP network both these paradigms seem to be present simultaneously, as can be observed in detail by extracting its community structure [18]. This is done by obtaining the community partition of the network which maximizes the *modularity* 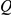 of the network, defined as

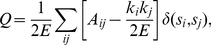
(26)


**Table 2:** The eleven keys with the largest number of signatures in the network, their respective in-degree 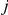, out-degree 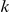, average in-degree of the nearest out-neighbours 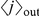, clustering coefficient 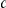, and date of creation.

Key ID	Name	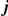	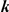	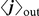	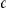	Date
D2BB0D0165D0FD58	CA Cert Signing Authority (Root CA) <gpg@cacert.org>	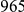	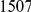	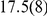	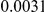	2003-07-11
2F951508AAE6022E	Karlheinz Geyer (TUD) <geyerk.fv.tu@nds.tu-darmstadt.de>	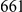	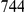	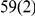	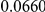	2004-12-07
DBD245FCB3B2A12C	ct magazine CERTIFICATE <pgpCA@ct.heise.de>	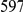	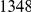	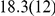	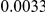	1999-05-11
69D2A61DE263FCD4	Kurt Gramlich <kurt@skolelinux.de>	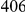	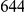	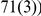	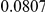	2002-10-17
948FD6A0E10F502E	Marcus Frings <protagonist@gmx.net>	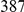	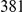	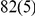	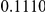	2002-03-22
29BE5D2268FD549F	Martin Michlmayr <tbm@cyrius.com>	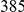	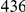	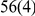	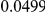	1999-08-04
566D362CEE0977E8	Jens Kubieziel <jens@kubieziel.de>	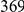	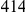	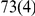	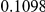	2002-08-23
3F101691D98502C5	Elmar Hoffmann <elho@elho.net>	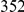	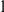	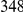	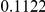	2005-02-17
957952D7CF3401A9	Elmar Hoffmann <elho@elho.net>	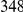	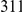	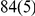	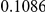	2005-02-17
CE8A79D798016DC7	Josef Spillner <josef@coolprojects.org>	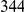	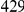	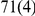	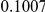	2001-05-22
89CD4B21607559E6	Benjamin Hill (Mako) <mako@atdot.cc>	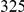	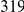	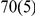	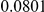	2000-07-13

where 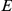 is the total number of edges, 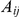 is the adjacency matrix of the network, 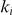 is the degree of vertex 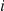, 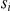 is the community label of vertex 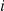 and 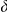 is the Kronecker delta. According to this definition, a partition with high values of 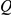 is possible for networks with densely-connected groups of vertices, with fewer connections between different groups. The maximum value of 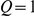 is achieved only for "perfect" partitions of extremely segregated communities. We note that the above definition is meaningful only for *undirected* graphs, and thus we apply it to the undirected version of PGP network, where the direction of the edges is ignored. We used the method of Reichardt et al [21] to obtain the best partition, which resulted in modularity value of 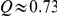. As a comparison, we computed the modularity for a shuffled version of the network, where the edges were randomly placed, but the in/out-degrees of the vertices were preserved, which resulted in the significantly smaller value 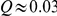. The distribution of community sizes seems to have a power-law tail with exponent 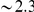 (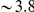 for the shuffled network), characterizing a scale-free structure. By isolating the individual communities, one can clearly see strong differences between those in the vicinity of the certificate authorities and “regular” communities. In Fig. 9 is shown two representative examples of these two types of communities: On top is the community around the CAcert.org certificate authority, and is composed of 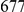 keys, with an average 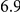 signatures per key. Its in/out-degree distributions are shown on the side, from which the large discrepancy between the most central vertex and the rest of the community can be observed. The colors on the vertices correspond to the Top-Level Domain (TLD) of the email addresses associated with each key, and serve as a coarse indication of the geographical proximity of the individuals. For the community containing CAcert.org, a high degree of geographical heterogeneity is present. This is corroborated also by the fact that there are fewer direct edges between individuals. On the bottom of Fig. 9 it is shown a community composed almost exclusively of keys with Austrian email addresses (.at TLD) which show a completely different pattern, lacking any central authority. It is smaller, with 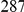 keys, but denser, with 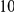 signatures per key. This pattern is repeated for most of the largest communities in the graph. Some non-centralized communities have a broader in/out-degree distribution than the Austrian community, but only those associated with certificate authorities display a centralized pattern such as in the top of Fig. 9.

**Figure 9:**
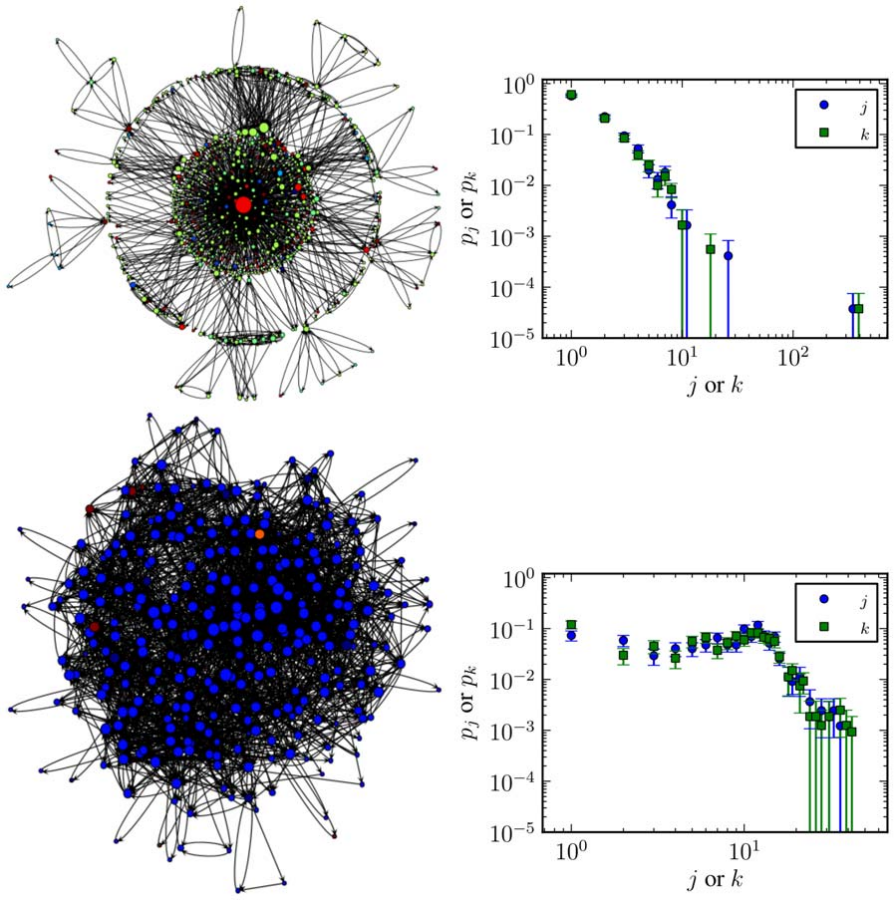
Two example communities of the PGP network, and their in- and out-degree distributions. The colors on the vertices correspond to the top-level domain (TLD) of the email addresses. **Top:** Community containing the CACert.org certificate authority. **Bottom:** Community composed mostly of Austrian email addresses (.at TLD).

We now turn to the trust propagation on the PGP network.

#### 2.2 Trust transitivity

In order to properly investigate trust transitivity in the PGP network, it is necessary to know the direct trust values associated with each signature, which indicate the level of scrutiny in the key verification process. The OpenPGP standard [13] defines four trust “classes” for signatures, according to the degree of verification made. Unfortunately, these classes are universally ignored, and most signatures fall into the “generic” class, from which no assertion can be made. Since the actual level of verification of the keys is in fact unknown, we will investigate hypothetical situations which represent different strategies the PGP users may use to verify keys. In the last section we have shown that the network is composed of different connection patterns: community clusters and centralized trust authorities. Depending on how these connection patterns are judged more trustworthy, the values of transitive trust will be different. Here we will consider three possible scenarios: 1. Random distribution, 2. Authority-centered trust, and 3. Community-centered trust. In all situations we will consider that all signatures have the same trust value of 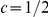, except for a fraction 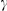 of edges which have absolute trust 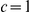, which is selected as follows for each situation:

1. *Random:* The 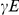 edges are chosen randomly among all 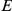 edges.
2. *Authority-centered:* The 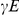 edges with the largest *betweenness* [22] 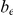 are chosen, which is defined as

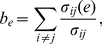
(27)where 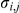 is the number of shortest paths from vertex 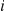 to 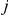, and 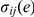 is the number of these paths which contain the edge 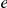. This distribution favours edges adjacent to nodes with high in/out-degree, and also edges which bridge different communities.
3. *Community-centered:* The 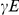 edges with the largest *edge clustering* 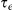 are chosen, which is defined as

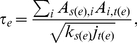
(28)where 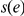 and 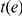 are the source and target vertices of edge 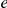, 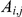 is the adjacency matrix, and 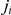 and 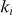 are the in- and out-degrees of vertex 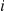, respectively. This quantity measures the density of out-neighbours of the 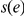 which are also in-neighbours of 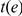, and simultaneously the density of in-neighbours of 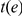 which are out-neighbours of 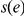 (this definition is equivalent to a normalized version of the *edge multiplicity* defined in [23]–[25]). This distribution favours edges with belong to densely-connected communities. For instance, the edges of a *clique* (i.e. a complete subgraph) will all have the value 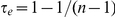, where 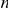 is the size of the clique, which will approach the maximum value 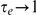 for a sufficiently large clique size.

**Figure 10:**
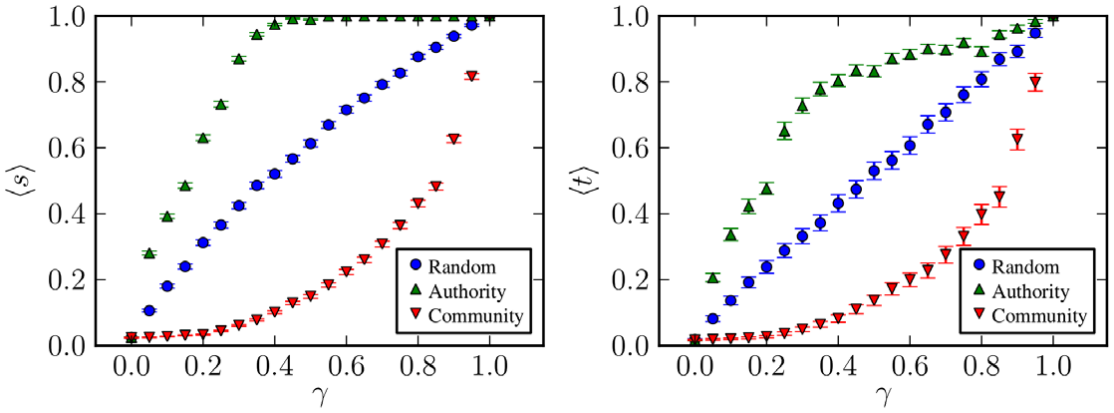
Average best trust 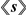 and pervasive trust 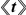, as a function of the fraction of edges with absolute trust 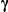, for the PGP network. The different curves correspond to the different trust distribution scenarios described in the text.

**Figure 11:**
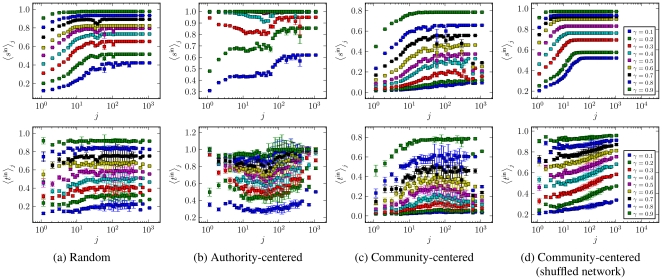
Average best in-trust 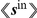 and pervasive in-trust 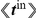, as a function of the in-degree 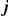 and the fraction of edges with absolute trust 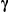, for the PGP network. The different plots correspond to the different trust distribution scenarios described in the text: (a) Random distribution, (b) authority-centered distribution and (c) community-centered distribution. The plots (d) correspond to a community-centered distribution, done on a shuffled version of network, with the same degree sequence.

In Fig. 10 it is shown the average best trust transitivity, Eq. 1 and average pervasive trust Eq. 7 for the PGP network, as a function of 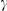 according to the different approaches. We note that no discontinuous transition is seen. This is probably due to the numerous topological differences from purely random networks (i.e. correlations, reciprocity, community structure, clustering), as described previously, as well as relatively small size of the network, all of which may cause the transition to disappear. The authority-centered trust leads to significantly higher values of 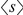 and 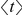, and the community-based distribution to the lowest values. This is expected, since distributing trust according to the edge betweenness essentially *optimizes* trust transitivity, putting the highest values along the shortest paths between vertices. The community-centered approach does exactly the opposite, favoring intra-community connections, and results in the lowest values of average trust. Thus, favoring the hubs and authorities is clearly more *efficient*, if the objective is solely to increase the average trust in the network. However, pure efficiency may not be what is desired, since it relies in the opinion of a much smaller set of vertices, which eases the job of dishonest parties, which need only to convince these vertices in order to be trusted by a large portion of the network. Some of these issues become more clear by observing how nodes with different in-degrees receive trust with each of these strategies, as show in Fig. 11. More specifically, what is shown is the average pervasive and best *in-trust* for vertices with different in-degrees, which are respectively defined as 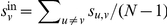 and 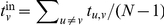, for a given vertex 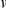. For a random distribution of direct trust, the vertices with higher in-degree receive a natural bias in the values of average best in-trust, 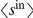, since the shortest paths leading to them tend to be smaller. But the fair nature of the definition of 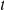 compensates for this, and the values of 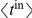 are almost independent of the in-degree of the vertices. The highly connected nodes become more trusted only with the authority-centred approach. Interestingly, in this situation the nodes with the *smallest* in-degrees also receive a large value of trust, since most of them are “fringe” nodes connected only with the hubs (see Fig. 7). The vertices with intermediary in-degrees are thus left in the limbo, and are in effect *penalized* for their community pattern. The almost symmetrically opposite situation is obtained with the community-centered trust distribution, where both the vertices with smallest and largest in-degrees receive the smallest trust values, and the intermediary nodes are judged more trustworthy due to their strong communities. We note that this effect is not due simply to the way the values of trust are distributed, but depend strongly on the existence of communities in the network. This is evident when the same trust distribution is applied to a shuffled version of the network, with the same in/out-degree sequence, as is shown in Fig. 11. For such a network, the community structure disappears, and the highly connected nodes come again in the lead.

## Discussion

We investigated properties of trust propagation on network based on the notion of trust transitivity. We defined a trust metric, called *pervasive trust* which provides inferred trust values for pairs of nodes, based on a network of direct trust values. The metric extends trust transitivity to the situation where multiple paths between source and target exist, by combining the best trust transitivity to the in-neighbours of a given target node, and their direct trust to the target. The trust values so-obtained are unbiased, personalized and well defined for any possible network topology. Equipped with this metric we analyzed the conditions necessary for global trust propagation in large systems, using random networks with arbitrary in/out-degree distributions as a simple model. We analytically obtained the average best trust transitivity (as well as pervasive trust) as a function of the fraction 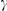 of edges with *absolute* trust 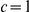. We found that there is a specific value of 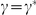, below which the average trust is always zero. For 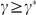 the average value jumps discontinuously to a positive value.

Using the defined trust metric, we investigated trust propagation in the Pretty Good Privacy (PGP) network [4], [5]. We gave an overview of the most important topological and dynamical features of the PGP network, and identified mixed connectivity patters which are relevant for trust propagation: namely the existence of trust authorities and of densely-connected non-centralized communities. Based on these distinct patterns, we formulated different scenarios of direct trust distribution, and compared the average inferred trust which results from them. We found that an authority-centered approach, where direct trust is given preferentially to nodes which are more central, leads to a much larger average trust, but at the same time benefits nodes at the fringe of the network, which are only connected to the authority hubs, and for which no other information is available. Symmetrically, a community-centered approach, where edges belonging to densely-connected communities are favoured with more trust, results in less overall trust, but both the fringe nodes and the authorities receive significantly less trust than average. These differences are not simply due to the different ways the direct trust is distributed, but rather to the fact that the dense communities and the trust authorities are somewhat segregated. These differences illustrate the advantages and disadvantages of both paradigms of trust propagation, which seem to be coexist in the PGP network. It also serves as an insightful example of how dramatically the direct trust distribution can influence the inferred trust, even when the underlying topology remains the same.

In this work, we have concentrated on static properties of trust propagation. However most trust-based systems are dynamic, and change according to some rules which are influenced by the trust propagation itself. One particularly good example is market dynamics [1], [2] where sellers (or borrowers) do not perform well if they have a poor track record, which will be partially influenced by trust. Thus, it remains to be seen how trust transitivity can be carried over to such types of models, and what role it plays in shaping their dynamics.
